# Hierarchy of evidence referring to the central nervous system in a high-impact radiation oncology journal: a 10-year assessment. Descriptive critical appraisal study: Hierarquia de evidência referindo-se ao sistema nervoso central em um jornal de radioterapia de alto impacto: uma avaliação de 10 anos. Estudo descritivo de avaliação crítica

**DOI:** 10.1590/1516-3180.2014.8792210

**Published:** 2015-07-03

**Authors:** Fabio Ynoe Moraes, Lorine Arias Bonifacio, Gustavo Nader Marta, Samir Abdallah Hanna, Álvaro Nagib Atallah, Vinícius Ynoe Moraes, João Luis Fernandes Silva, Heloísa Andrade Carvalho

**Affiliations:** I MD. Physician, Department of Radiation Oncology, Hospital Sírio-Libanês, São Paulo, Brazil.; II MD. Radiation Oncologist, Department of Radiation Oncology, Hospital Sírio-Libanês, São Paulo and Radiation Oncologist, Department of Radiation Oncology, Instituto do Câncer do Estado de São Paulo, São Paulo, Brazil.; III MD, PhD. Radiation Oncologist. Department of Radiation Oncology, Hospital Sírio-Libanês, São Paulo, Brazil.; IV MD, PhD. Full professor and head of the Discipline of Emergency Medicine and Evidence-Based Health of Universidade Federal de São Paulo - Escola Paulista de Medicina. Director of the Brazilian Cochrane Center, São Paulo, Brazil.; V MD. Orthopedic Surgeon, Department of Orthopedics and Hand Surgery, Universidade Federal de São Paulo - Escola Paulista de Medicina, São Paulo, Brazil.; VI MD. Radiation Oncologist and Head of the Department of Radiation Oncology, Hospital Sírio-Libanês, São Paulo, Brazil.; VII MD, PhD. Radiation Oncologist, Radiotherapy Service, Institute of Radiology, Hospital das Clínicas, Faculdade de Medicina da Universidade de São Paulo, São Paulo, Brazil, and Radiation Oncologist, Department of Radiation Oncology Hospital Sírio-Libanês, São Paulo, Brazil.

**Keywords:** Radiotherapy, Central nervous system neoplasms, Epidemiologic methods, Research design, Evidence-based medicine, Radioterapia, Neoplasias do sistema nervoso central, Métodos epidemiológicos, Projetos de pesquisa, Medicina baseada em evidências

## Abstract

**CONTEXT AND OBJECTIVE:**

To the best of our knowledge, there has been no systematic assessment of the classification of scientific production within the scope of radiation oncology relating to central nervous system tumors. The aim of this study was to systematically assess the status of evidence relating to the central nervous system and to evaluate the geographic origins and major content of these published data.

**DESIGN AND SETTING:**

Descriptive critical appraisal study conducted at a private hospital in São Paulo, Brazil.

**METHODS:**

We evaluated all of the central nervous system studies published in the journal Radiotherapy & Oncology between 2003 and 2012. The studies identified were classified according to their methodological design and level of evidence. Information regarding the geographical location of the study, the institutions and authors involved in the publication, main condition or disease investigated and time of publication was also obtained.

**RESULTS:**

We identified 3,004 studies published over the 10-year period. Of these, 125 (4.2%) were considered eligible, and 66% of them were case series. Systematic reviews and randomized clinical trials accounted for approximately 10% of all the published papers. We observed an increase in high-quality evidence and a decrease in low-quality published papers over this period (P = 0.036). The inter-rater reliability demonstrated significant agreement between observers in terms of the level of evidence.

**CONCLUSIONS:**

Increases in high-level evidence and in the total number of central nervous system papers were clearly demonstrated, although the overall number of such studies remained relatively small.

## RESUMO

**CONTEXTO E OBJETIVO:**

Até onde sabemos, não há avaliação sistemática da classificação da produção científica no âmbito da radioterapia de tumores de sistema nervoso central. O objetivo deste estudo foi avaliar sistematicamente o estado das evidências relativas ao sistema nervoso central e avaliar origem geográfica e a temática envolvida nestas publicações.

**TIPO DE ESTUDO E LOCAL:**

Estudo descritivo de avaliação crítica, realizado em um hospital privado em São Paulo, Brasil.

**MÉTODOS:**

Foram avaliados todos os estudos publicados em sistema nervoso central na revista Radiotherapy & Oncology, entre 2003 e 2012. Os estudos identificados foram classificados de acordo com o desenho metodológico e nível de evidência. Informações sobre a localização geográfica do estudo, instituições e os autores envolvidos nas publicações, a principal condição ou doença estudada e o período de publicação também foram obtidos.

**RESULTADOS:**

Foram identificados 3.004 estudos publicados no período de 10 anos. Destes, 125 (4,2%) foram considerados como elegíveis, e 66% destes eram séries de casos. As revisões sistemáticas e ensaios clínicos randomizados foram responsáveis por cerca de 10% de todas as publicações. Observou-se um aumento das evidências de alta qualidade e uma diminuição das publicações de baixa qualidade durante o período (P = 0,036). A confiabilidade entre avaliadores demonstrou concordância significativa para níveis de evidência.

**CONCLUSÕES:**

Um aumento nas evidências de alto nível, assim como no número absoluto de artigos em sistema nervoso central foi claramente demonstrado, apesar de o número global ser ainda relativamente pequeno.

## INTRODUCTION

Evidence-based medicine has become essential to clinical and research actions since it was formally proposed in 1990.^1^ The importance of evidence-based medicine concepts was highlighted in an article published in the British Medical Journal in 2007, in which the editors described the emergence of evidence-based medicine as one of the 15 most important milestones since the foundation of the British Medical Journal (1870).^2^^,^^3^ Henceforth, critical evaluation of evidence has become an important tool for assessing research quality and progress. Clinical research can be classified into levels of evidence, which are based on evaluating and interpreting evidence. The level of evidence is closely related to the likelihood that a piece of research will produce valid and reliable results.

Radiotherapy is no different in this regard. The pursuit of the best evidence is changing and is beginning to follow the trends reported in the 1990s.^4^ As an example, conducting a quick Medline search associating “randomized trials” and “radiation oncology”, 211, 144, 27 and 5 studies for the years 2012, 1996, 1981 and 1970 are identified, respectively. This finding demonstrates the evolution and intensification of research applied to radiotherapy, with a 40-fold increase in publications, over this time period.

Moreover, high-quality studies play a fundamental role in medical journals. From a broader perspective, the methodological quality and level of evidence of published articles are important determinants of how many times an article is cited, which therefore affects the impact factor of that journal and can also play a major role in the clinical transfer of knowledge.^5^^,^^6^ This has become an essential aspect of the evaluation of scientific journals.^6^ In 2003, prominent journals began to use evidence hierarchies to rank the published studies.^7^^,^^8^ As a result, evidence-based medicine concepts were adopted by the conferences and symposia of the main specialties. Following this paradigm, great efforts have been applied within radiation oncology to follow the evidence-based medicine trend. Nevertheless, to date, there has been no systematic assessment of the quality of scientific production in several areas of radiation oncology.^4^^,^^5^

## OBJECTIVE

The aim of this study was to identify central nervous system studies published in Radiotherapy & Oncology (Elsevier Ireland) over the last decade (2003-2012), classify the type of study and evidence levels according to evidence-based medicine criteria and observe the inter-rater agreement in the classification of the studies included.

## METHODS

Using electronic databases, two researchers independently evaluated all studies published in all editions of the major European radiation oncology-specific journal (Radiotherapy & Oncology, Elsevier Ireland, accessed at http://www.thegreenjournal.com) between 2003 and 2012. This journal was chosen because it is important in the field of radiation oncology field; it is indexed in at least one major international database; and it is, so far, the radiation oncology journal with the highest impact factor. We conducted a descriptive critical appraisal study.

Studies in this journal were initially screened based on their titles and were classified as eligible, potentially eligible or not eligible. The sole inclusion criterion was that they needed to be clinical studies relating to the central nervous system that were published between 2003 and 2012. Thus, presence of the following topics in the title counted for this initial screening: metastatic central nervous system; low-grade glioma; high-grade glioma; pediatrics and central nervous system (medulloblastoma, ependymoma or astrocytoma); central nervous system lymphoma; benign tumors (meningioma, schwannoma or arterial venous malformations); spinal cord, orbital and skull-base tumors; and experimental central nervous system studies. After this initial screening, the selected studies (eligible and potentially eligible) were first reassessed using their abstracts and then by using their full texts. All studies relating only to dosimetry were excluded. A third evaluator resolved any disagreements.

The studies thus identified were assessed by two examiners and were subsequently classified according to the methodological design: 1. systematic reviews; 2. randomized or non-randomized clinical trials; 3. cohort studies; 4. case-control studies; 5. case series; and 6. basic science studies. The studies were also classified according to their level of evidence using the guidelines of the Oxford Centre for Evidence-based Medicine: systematic reviews of randomized clinical trials, level I; randomized clinical trials, level II; cohort and case-control studies, level III; case series, level IV; and narrative reviews and other designs, level V. This is a widely used classification method that has been adapted for use within the radiation oncology literature.^9^ This categorization was done after reading the full texts of the eligible studies.

For all the studies ultimately included, we also obtained information regarding the geographical location at which the study was performed, institutions/departments and authors involved in the publication, main condition studied, main disease investigated and time of publication. We also examined the productivity relating to radiotherapy for the central nervous system in each department over the 10-year period covered by this analysis. The following parameters were stratified using the following parameters: time of publication (period 1: 2003-2007; and period 2: 2008-2012); geographical location; level of evidence; and Scientific Journal Rankings index (www.scimagojr.com). This index measures the impact that a single published paper has, and hence the scientific influence of an average article in a journal. It thus expresses the extent to which an average journal article is central to the global scientific discussion.

### Statistical analysis

The assumption of normal distribution in the sample was assessed using the Kolmogorov-Smirnov test. Cohen’s kappa test was used to assess reliability and to evaluate the internal consistency of the inter-rater classifications. The magnitude of agreement was determined based on the proposal of Landis and Koch: I. < 0, no agreement; II. 0 to 0.20, slight agreement; III. 0.21 to 0.40, fair agreement; IV. 0.41 to 0.60, moderate agreement; V. 0.61 to 0.80, significant agreement; and VI. 0.81 to 1.00 almost perfect agreement.^10^^,^^11^ The chi-square test was used to evaluate the proportions of papers at evidence levels I, II and III between the two periods. We considered P-values from two-sided tests < 0.05 to be statistically significant.

## RESULTS

**Figure 1.**
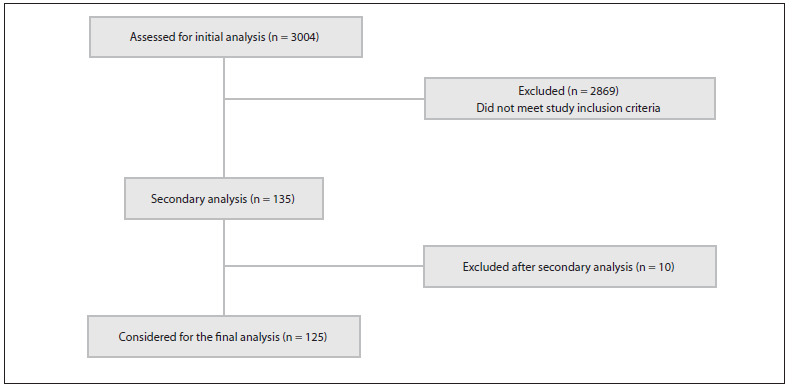
Flow diagram.

We identified 3,004 studies published over the 10-year period evaluated. Of these, 135 were initially selected (central nervous system disease), from which 10 were then excluded. Thus, 125 studies (4.2%) were considered eligible and were included in this analysis (Figure 1). There was an average of 300.4 publications per year during the study period (which included an average of 13.5 publications per year relating to the central nervous system). We noted an absolute increase in the number of published papers of 33% overall and 41% in relation to the central nervous system, from period 1 to period 2 (Table 1).

**Table 1.**
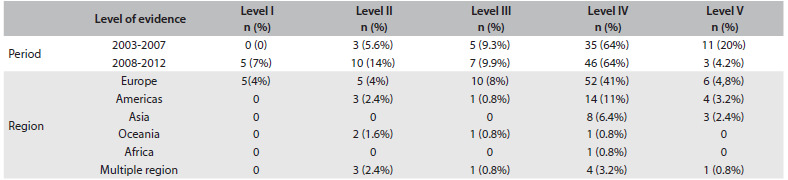
Frequencies of the hierarchy of evidence, grouped according to the period and region of origin

Table 2 shows the distribution of the central nervous system studies according to the geographical location at which they were conducted. European studies accounted for more than 60% of the published data over the entire period and, in comparison with the rest of the world, this difference was statistically significant (P = 0.0306).

**Table 2.**
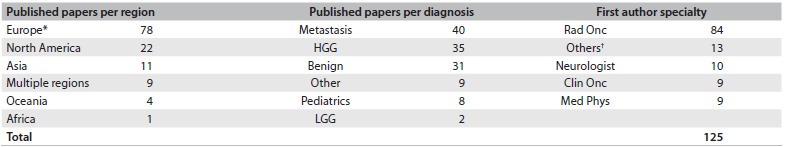
Central nervous system papers published, according to region, diagnosis and first author over the 10-year period

*Chi-square test, P = 0.03 (when comparing Europe versus all other regions). ^†^Others: radiologist, palliative care specialist, epidemiologist, nuclear medicine specialist, pediatrician and not specified. Abbreviations: HGG = high-grade glioma; LGG = low-grade glioma; Rad Onc = radiation oncologist; Clin Onc = clinical oncologist; Med Phys = medical physicist.

Stratification according to disease classification showed that the majority (74%) of the studies were related to central nervous system metastasis, followed by high-grade gliomas and benign tumors (Table 2).

We also noted that the average numbers of authors and departments involved in the studies were 6.86 and 3.29, respectively; 67% of the first authors were radiation oncologists (Table 2), and 85% of the first authors only had a single article published in Radiotherapy & Oncology as the first author. Twelve institutions (University Hospital of Heidelberg, Institute of Southern Switzerland, University Hospital Zurich, University Hospital Groningen, Tata Memorial Hospital, University of Wisconsin Medical School, McGill University Health Center, ‘‘S. Maria” Hospital, VU University Medical Center, St. Jude Children’s Research Hospital, Ludwig-Maximilians-University Munich and San Raffaele Scientific Institute) (13.3% of the total) were responsible for 40 published papers (32% of the total) and 78 institutions were responsible for the other 68% of publications over this 10-year period.

Among these studies, 66.5% were case series (prospective and retrospective; number published (n) = 81 articles); 8% were prospective controlled studies (not randomized) (n = 10); 1% were cohort studies (n = 1); 1% were case-control studies (n = 1); 6% were cross-sectional studies (n = 8); and 3% were review articles (n = 5). Systematic reviews (n = 5) and randomized clinical trials (n = 7) accounted for approximately 10% of all the published papers. Other studies, which included case reports, were responsible for 5% of the publications (n = 7). In analyzing the level of evidence according to year, we observed that there were greater numbers of published papers with evidence levels I, II and II and lower numbers with evidence levels IV and V in period 2 (2008-2012) than in period 1 (2003-2007) (P = 0.036).^9^

The Scientific Journal Rankings index showed average values of 1.38 and 1.89 for periods 1 and 2, respectively. This higher index for period 2 presented a tendency towards a statistically significant difference in relation to period 1 (P = 0.0528). The inter-rater reliability for the classification of study type according to the kappa statistic demonstrated significant agreement between the observers (kappa = 0.69).

## DISCUSSION

In this 10-year single-journal analysis, we found that the studies published within the scope of the central nervous system increased in quality and number, although significant representation in the journal Radiotherapy & Oncology is still lacking (< 4.5% of published papers). We also found that case series (retrospective and prospective) represented the majority of central nervous system papers published in this journal. Furthermore, level of evidence was found to be a reproducible tool, and secondary tumor (metastasis) research was well represented in this journal. The major strength of our data is that they represent, to the best of our knowledge, an original study with a representative period of evaluation in a single journal. Moreover, this analysis was based on formal and systematic data-gathering and evaluation, and our results present sequential assessment, including formal statistical analysis and inter-reliability analysis based on Cohen’s kappa test.

In the United States, according to the national database, primary central nervous system tumors account for less than 3% of all diagnosed neoplasms.^12^ Similarly, primary and metastatic central nervous system tumors, together with benign central nervous system diseases, represent vast opportunities for treatment improvements, with implementation of new markers and prognostic factors. This is an important field for radiotherapy research, including newer approaches using stereotactic radiosurgery. In the field of central nervous system tumors, radiotherapy plays a major role in the management of almost all types of malignant brain tumors. Moreover, a high level of evidence can play a major role in treatment decisions.

Among patients diagnosed with cancer (all anatomical sites), approximately 54% of all of them will require some radiation treatment during their lifetime, and 12% will require re-irradiation.^13^ Based on evidence-based guidelines, the central nervous system shows a highly recommended overall optimal radiotherapy utilization rate (approximately 92-93%).^13^ In a comprehensive analysis in which the objective was to estimate the ideal proportion of patients with newly diagnosed central nervous system neoplasms who could benefit from external-beam radiotherapy, most of the recommendations were based on evidence level III.^14^ For tumors at other sites, such as cervical tumors (62% presenting levels IV/V), hematological malignancies (majority presenting level III) and gastrointestinal tumors (mainly presenting levels II/III), the clinical recommendation for radiotherapy was based on low/moderate level of evidence.^15^^,^^16^^,^^17^^,^^18^ Based on our analysis, most of the studies presented low-level evidence, such as prospective and retrospective case series (66%). Higher evidence levels such as systematic reviews and randomized clinical trials represented only approximately 10% of all central nervous system published papers. In comparison with case series, randomized trials involve greater numbers of ethical issues and higher costs. The incidence of primary central nervous system tumors, combined with the tendency to treat them in large specialized centers, may explain the findings of this study. It also needs to be taken into consideration that the journal Radiotherapy & Oncology accepts physical contributions, dosimetry studies, molecular biology assays and other types of non-formal clinical publications. In addition to the important role of such articles with regard to development of radiation oncology, they are classified as presenting evidence levels IV or V according to the Oxford Criteria.^9^ Nevertheless, there was an increase in the evidence level of published central nervous system articles (in our data) over the years, particularly in the more recent period.

In a manner similar to our study, Yarascavitch et al. (at McMaster University, Hamilton, Canada) quantified the level of evidence in 660 eligible articles in the neurosurgical literature in order to determine the changes over time and the predictive factors for higher-level evidence.^19^ Levels I and II accounted for only 1 in 10 neurosurgical clinical papers in top journals, and papers with larger sample sizes were significantly associated with higher level of evidence. These authors concluded that there is a need for better evidence in papers published within this field and that patient management and the publication of prospective studies may be improved by education and the adoption of level of evidence. In addition, other analyses have suggested that improvements in the evidence of published studies are possible, although most of the published papers in many fields remain at evidence level IV.^6^^,^^20^^,^^21^^,^^22^^,^^23^^,^^24^

Similarly, it is important to note that level of evidence can be correlated with journal impact factor and that increasing numbers of studies with high-level evidence have been observed in palliative and orthopedic settings.^22^^,^^25^^,^^26^ This finding emphasizes that there is an urgent need to expand the data relating to evidence-based oncology. Regarding the origin of the articles included in this study, in comparison with the rest of the world, Europe showed the largest number of published papers within the field of the central nervous system (60%). Because Radiotherapy & Oncology is a journal based and supported in Europe, this could represent a bias of our study. However, we did not find any previous data evaluating the regions in which central nervous system radiation oncology publications are produced. This may reflect the tendency to centralize more specialized procedures in reference centers that can better address central nervous system diseases. We also found that most authors published just one article as first author and then either ceased to be involved in research or acquired greater independence and became senior researchers. However, this was not evaluated in the present study. In addition, Morgan et al. evaluated scientific production relating to radiation oncology in the United States^27^ and observed that there was an average of one peer-reviewed first-author publication and one first-author abstract presentation at an annual meeting of the American Society for Therapeutic Radiology and Oncology (ASTRO) per resident during their four years of training. We consider that this sort of analysis is important for future studies and for knowledge of referral centers for future postgraduate training.

The limitations of the current analysis lie in the fact that central nervous system articles may not be well represented in the journal chosen for analysis here because other radiation oncology journals that were not included in the electronic search also publish articles relating to the central nervous system. In addition, specific journals and higher-impact journals may account for significant numbers of published papers relating to the central nervous system. These were not assessed in the present analysis but may have had an impact on the data presented. Furthermore, a wider search of the literature might lead to a more optimistic outlook regarding the proportion of high-quality studies.

In this study, a training workshop on manuscript classification was conducted initially. The Oxford system of levels of evidence seemed to be a feasible instrument for evaluating studies, with a significant degree of consistency.^9^ These findings and those in other studies emphasize the importance of specific training for individuals who are responsible for determinations relating to the quality of evidence.^28^ Finally, our study represents a possible landmark for future studies and other evidence-based assessments on the central nervous system within the field of radiation oncology research. Moreover, the present study may result in new research opportunities, such as assessment of the internal and external validity of other study features and evaluations on high-impact and specialized journals. In particular, it would be interesting to evaluate how radiation oncologists manage and comprehend evidence-based medicine.

## CONCLUSION

Both the number and the level of evidence of published papers relating to the central nervous system have increased in the journal Radiotherapy & Oncology. However, between 2003 and 2012, central nervous system papers still represented less than 5% of all publications with evidence levels I and II and accounted for only 10% of the papers published in this specific journal. Our analysis also encourages the use of levels of evidence, which is a useful tool with significant inter-rater reliability.
